# Oropharyngeal cancer: United Kingdom National Multidisciplinary Guidelines

**DOI:** 10.1017/S0022215116000505

**Published:** 2016-05

**Authors:** H Mehanna, M Evans, M Beasley, S Chatterjee, M Dilkes, J Homer, J O'Hara, M Robinson, R Shaw, P Sloan

**Affiliations:** 1 Institute of Head and Neck Studies and Education, University of Birmingham, Birmingham, UK; 2 Velindre Cancer Centre, Cardiff, UK; 3 Bristol Haematology and Oncology Centre, University Hospitals Bristol NHS Foundation Trust, Bristol, UK; 4 Tata Medical Center, Kolkata, India; Newcastle University, Newcastle upon Tyne, UK; 5 ENT Department, Barts and The London NHS Trust, London, UK; 6 Department of Otolaryngology-Head and Neck Surgery, Manchester Royal Infirmary and Christie Hospital, University of Manchester, Manchester, UK; 7 Department of Otolaryngology, The Newcastle upon Tyne Hospitals NHS Foundation Trust, Newcastle upon Tyne, UK; 8 School of Dental Sciences, Newcastle University, Newcastle upon Tyne, UK; 9 Department of Molecular and Clinical Cancer Medicine, Liverpool CR-UK Centre, Institute of Translational Medicine, University of Liverpool, Aintree University Hospitals NHS Foundation Trust, Liverpool, UK; 10 Department of Cellular Pathology, The Newcastle upon Tyne Hospitals NHS Foundation Trust, Oral Health Research Group, Newcastle University, Newcastle upon Tyne, UK

## Abstract

This is the official guideline endorsed by the specialty associations involved in the care of head and neck cancer patients in the UK. There has been significant debate in the management of oropharyngeal cancer in the last decade, especially in light of the increased incidence, clarity on the role of the human papilloma virus in this disease and the treatment responsiveness of the human papilloma virus positive cancers. This paper discusses the evidence base pertaining to the management of oropharyngeal cancer and provides recommendations on management for this group of patients receiving cancer care.

**Recommendations:**

• Cross-sectional imaging is required in all cases to complete assessment and staging. (R)

• Magnetic resonance imaging is recommended for primary site and computed tomography scan for neck and chest. (R)

• Positron emission tomography combined with computed tomography scanning is recommended for the assessment of response after chemoradiotherapy, and has a role in assessing recurrence. (R)

• Examination under anaesthetic is strongly recommended, but not mandatory. (R)

• Histological diagnosis is mandatory in most cases, especially for patients receiving treatment with curative intent. (R)

• Oropharyngeal carcinoma histopathology reports should be prepared according to The Royal College of Pathologists Guidelines. (G)

• Human papilloma virus (HPV) testing should be carried out for all oropharyngeal squamous cell carcinomas as recommended in The Royal College of Pathologists Guidelines. (R)

• Human papilloma virus testing for oropharyngeal cancer should be performed within a diagnostic service where the laboratory procedures and reporting standards are quality assured. (G)

• Treatment options for T1–T2 N0 oropharyngeal squamous cell carcinoma include radical radiotherapy or transoral surgery and neck dissection (with post-operative (chemo)radiotherapy if there are adverse pathological features on histological examination). (R)

• Transoral surgery is preferable to open techniques and is associated with good functional outcomes in retrospective series. (R)

• If treated surgically, neck dissection should include levels II–IV and possibly level I. Level IIb can be omitted if there is no disease in level IIa. (R)

• If treated with radiotherapy, levels II–IV should be included, and possibly level Ib in selected cases. (R)

• Altering the modalities of treatment according to HPV status is currently controversial and should be undertaken only in clinical trials. (R)

• Where possible, patients should be offered the opportunity to enrol in clinical trials in the field. (G)

## Introduction and epidemiology

The incidence of oropharyngeal squamous cell carcinoma (OPSCC) is increasing significantly in developed countries.^1^ In the USA, the incidence increased by 22 per cent from 1.53 per 100 000 to 1.87 per 100 000 between 1999 and 2006, after showing no change between 1975 and 1999. The UK has seen a doubling of incidence between 1990 and 2006. There has been a further doubling in incidence between 2006 and 2010.

The increasing incidence of OPSCC is due to human papilloma virus (HPV) infection, with HPV-16 being the predominant subtype responsible. The proportion of cases with evidence of HPV infection has risen rapidly and HPV is now responsible for over 70 per cent of OPSCCs in Europe and the USA.^1^^,^^2^ The rise in HPV-related OPSCC has been called an ‘epidemic’ and is expected to continue.

## Clinical presentation

Patients often present with a painless neck lump, with few other symptoms. They may also complain of a sore throat or tongue, otalgia, pain and/or difficulty swallowing and/or a change in voice quality (hot potato voice).

## Assessment and staging

### Clinical examination

Flexible direct endoscopy of the upper aerodigestive tract is now available in virtually all ear, nose and throat clinics in the UK. It is vital for assessing the limits of spread, such as direct through and through invasion of the soft palate from anterior to posterior surfaces, the inferior extent of lateral pharyngeal wall tumours into the vallecula and pyriform fossa, and the superior extension of tonsillar cancers into the postnasal space and skull base.

### Imaging considerations

Cross-sectional imaging is required in all cases to complete assessment and staging. Magnetic resonance imaging (MRI) scanning with contrast is optimal for staging the primary tumour, particularly when assessing soft tissue spread, such as in the tongue base and/or body of the tongue.^3^^,^^4^ Computed tomography (CT) scanning may also be required, particularly to assess the extent of nodal disease and bony invasion, e.g. body of the mandible and skull base in tonsillar tumours and cervical spine in posterior pharyngeal wall tumours.

The presence of nodal metastases should be evaluated by CT or MRI in all patients. Ultrasound with or without needle biopsy should be carried out for all patients presenting with a neck lump and is an accurate method of staging nodal disease in experienced hands.

Distant metastases should be assessed by CT scanning of the chest and upper abdomen, to exclude metastatic disease to the lungs and liver.^3^ Magnetic resonance imaging scanning is not suitable for this due to the relatively slow acquisition process leading to movement artefact caused by breathing.

Fluoro-deoxy-glucose positron emission tomography combined with computed tomography (F-FDG PET–CT) scanning may be used to give additional staging information when it is available, particularly where staging is difficult clinically (e.g. patient with trismus) or where there is uncertainty on other imaging and/or equivocal findings that would preclude radical treatment. Positron emission tomography (PET) also has a role in the assessment of recurrent tumours and can detect recurrence at primary sites, neck nodes and/or distant metastases.

Supported by the results of the UK PET-Neck randomised controlled trial (RCT) study,^5^ F-FDG PET–CT scanning is now also recommended for the assessment of response approximately three months post-chemoradiotherapy, particularly in patients with advanced nodal disease. PET-CT guided active surveillance showed similar survival outcomes to the planned neck dissection arm, but resulted in considerably fewer neck dissections, and fewer complications, and was cost effective, supporting its use in routine practice.^5^

### Examination under anaesthetic and panendoscopy

Examination under anaesthetic and panendoscopy is strongly recommended to assess the extent and resectability of the primary tumour and to exclude second primaries, especially in hypopharynx and oesophagus. Examination under anaesthetic is mandatory if thorough endoscopic examination is not possible in the clinic as above and/or if no biopsy can be obtained.

**Recommendations**

• Cross-sectional imaging is required in all cases to complete assessment and staging (R)
• Magnetic resonance imaging is recommended for primary site and CT scan for neck and chest (R)
• Positron emission tomography combined with computed tomography scanning is recommended for the assessment of response after chemoradiotherapy, and has a role in assessing recurrence (R)
• Examination under anaesthetic is strongly recommended, but not mandatory (R)

### Pre-treatment staging

Pre-treatment staging for the primary tumour based on the tumour–node–metastasis classification (7th edition) for oropharyngeal tumours is shown in Box I.

BOX I **TNM STAGING FOR OROPHARYNGEAL SQUAMOUS CELL CARCINOMA**

• TX: Primary tumour cannot be assessed
• T0: No evidence of primary tumour
• Tis: Carcinoma in situ
• T1: Tumour 2 cm or less in greatest dimension
• T2: Tumour larger than 2 cm but 4 cm or less in greatest dimension
• T3: Tumour larger than 4 cm in greatest dimension or extension to lingual surface of epiglottis
• T4a: Tumour invades the larynx, deep/extrinsic muscle of tongue, medial pterygoid, hard palate or mandible
• T4b: Tumour invades lateral pterygoid muscle, pterygoid plates, lateral nasopharynx, or skull base or encases carotid artery

## Pathology

Formal tissue biopsy of the primary cancer is one of the cornerstones of the management pathway in oropharyngeal cancer. Tumours can be biopsied under local or no anaesthetic in the clinic. Otherwise, direct biopsy and staging under general anaesthetic is necessary.

In very few circumstances, a positive cancer diagnosis from fine needle aspiration (FNA) of involved nodes may suffice, provided the cytology result has been considered in conjunction with the clinical presentation and appropriate imaging at a head and neck cancer multidisciplinary team meeting. Such circumstances may arise in a person who is unfit to have an anaesthetic for an open biopsy and in whom local anaesthetic biopsies have not been successful. There is limited information on the reliability of p16 and HPV tests on FNA material and HPV testing is not currently routinely recommended on FNA samples.

The majority of oropharyngeal cancers are squamous cell carcinomas. It is recommended that they are reported according to The Royal College of Pathologists UK Guidelines for the histopathology reporting of mucosal malignancies of the pharynx (2013). Human papilloma virus testing is a core item for OPSCC to allow the stratification of treatment outcomes. Human papilloma virus status should be assessed using validated methods with appropriate controls. Human papilloma virus testing for oropharyngeal cancer should be performed within a diagnostic service where the laboratory procedures and reporting standards are quality assured. The immunohistochemical identification of over-expression of p16 protein is a useful screening method for HPV infection as HPV-associated carcinomas show strong nuclear and cytoplasmic expression of p16 in over 70 per cent malignant cells and p16-negative cases are almost certainly not HPV associated. Carcinomas showing p16 over-expression should have the presence of HPV confirmed by high-risk HPV DNA in situ hybridisation, if possible. Polymerase chain reaction analysis for HPV is not currently recommended in clinical practice as there is a risk of false positive results from formalin-fixed tissues.^6^

**Recommendations**

• Histological diagnosis is mandatory in most cases, especially for patients receiving treatment with curative intent (R)
• Oropharyngeal carcinoma histopathology reports should be prepared according to The Royal College of Pathologists Guidelines (G)
• Human papilloma virus testing should be carried out for all oropharyngeal squamous cell carcinomas as recommended in The Royal College of Pathologists Guidelines (R)
• Human papilloma virus testing for oropharyngeal cancer should be performed within a diagnostic service where the laboratory procedures and reporting standards are quality assured (G)

## Prognosis

Prognosis is dependent on stage at presentation as well as HPV status.^7^ The status of human papilloma virus is a strong and independent prognostic factor for survival, and HPV-positive OPSCC has a 58 per cent reduction in the risk of death compared with HPV-negative OPSCC (hazard ratio 0.42, 95 per cent; confidence interval  0.27–0.66), with 3 year overall survival rates of 82.4 per cent for HPV-positive disease compared with 57.1 per cent (*p* < 0.001) for HPV-negative disease.^8^ Factors including smoking, particularly current smoking,^9^^,^^10^ which may be a surrogate of genetic instability, and nodal stage, may influence prognosis in HPV-positive OPSCC. Several immunological markers have also been shown to correlate with prognosis and a UK study showed significant associations between the presence of tumour infiltrating lymphocytes and improved survival.^11^ Although there are no head-to-head comparisons of primary surgical *vs* non-surgical management for OPSCC, similar survival outcomes have been reported in studies of primary chemoradiotherapy and of surgery followed by post-operative radiotherapy (RT) and/or chemoradiotherapy, albeit there is a lack of prospective randomised trials of surgical management.^8^^,^^12^^–^^14^

To date, there is no evidence that patients with HPV-positive and HPV-negative OPSCC should be treated differently, outside of the context of randomised, controlled clinical trials. In view of the excellent prognosis from lower-risk HPV-positive disease, current and future UK studies (De-Escalate HPV, ISRCTN33522080 and PATHOS, UKCRN ID 18645) will investigate whether reduced intensity treatment can maintain favourable outcomes but reduce acute and late toxicity for patients. On the other hand, because HPV-negative and higher risk HPV-positive patients have a poorer prognosis, future trials (CompARE, ISRCTN41478539) will investigate whether escalating treatment will result in better outcomes for these patients.

## Management

### Early (T1–T2 N0) oropharyngeal carcinoma

#### General principles of management

Early stage (T1–T2 N0 M0) oropharyngeal carcinoma should ideally be treated with single modality therapy, either primary surgery or RT. There are no high-quality comparative studies of the two treatment modalities within the same population. Retrospective case series demonstrate five-year disease-specific survival rates of 81–100 per cent for primary surgery^15^(with adjuvant therapy where appropriate) and 77–89 per cent for primary RT, with surgical salvage.^16^ Treatment decisions are made based on the size and position of the tumour overall functional deficit.

#### Surgical management of early (T1–T2 N0) oropharyngeal cancer

Surgery for T1–T2 N0 OPSCC should usually be carried out transorally, either by transoral laser microsurgery (TLM) or transoral robotic surgery (TORS). Oncologic results after transoral resection of the oropharynx appear to be comparable to open surgery and good functional outcomes have been reported after transoral surgery in retrospective series.^17^ Open approaches are associated with increased severe morbidity and treatment complications and have now fallen out of favour for early stage disease.

During TLM, tumours are removed in several (at least two) planned pieces following trans-tumoural resection. This can cause difficulty in pathological scrutiny of the resected tissue to determine margins, which is compounded by laser artefact and difficulty in orientation. Representative marginal biopsies, taken from the peripheral mucosal resection margins and tumour bed can be carried out and examined pathologically to help rule out the presence of residual microscopic disease after TLM. In contrast to TLM, TORS involves *en bloc* removal of the tumour in the majority of cases. As a result, surgical margins can be more easily interpreted.

About 10–31 per cent of patients who are clinically T1–T2 N0 will have occult nodal disease. Therefore, patients having surgery to the primary should also undergo ipsilateral selective neck dissection. Surgery to the contralateral neck may also be considered in tumours arising at or very near the midline (in the soft palate, tongue base or posterior pharyngeal wall) in order to obtain pathological staging of the contralateral neck. Evidence suggests dissecting levels II, III and IV and possibly level I if there is anterior extension.^18^ Retrospective studies suggest that level IIb does not need to be dissected, as long as there are no findings pre-operatively of level IIa disease. For transoral resections, the neck dissection may be performed at the same time, or as a staged procedure, around two weeks before transoral resection of the primary. A staged approach may help prevent the development of a fistula if there is lateral pharyngeal wall transoral resection. Concomitant transoral resection and neck dissection can also be carried out and good results have been reported. In the latter, local muscle transposition (digastric or sternomastoid) can be performed to augment any defect and decrease risk of fistula. For any transoral resection of the oropharynx, ligation of the individual feeding vessels from the external carotid artery should be performed (ascending pharyngeal, lingual and facial branches) to limit the risk of potentially life-threatening haemorrhage. This should be done in any neck dissection performed as a prior staged procedure.

Although the goal for T1–T2 N0 disease should be single modality treatment, adjuvant RT and/or chemoradiotherapy may be required due to adverse pathological features for recurrence following surgery. Post-operative RT should be planned using the same principles as radical RT; a dose of 60 Gy in 30 fractions is typically recommended. Adjuvant treatment may affect functional outcomes following surgery.

#### Radical RT for early oropharyngeal cancer

Prior to RT, patients should undergo dietetic, speech and language therapy and dental review. A total dose equivalent of 70 Gy in 35 fractions is used in radical treatment. Hypofractionated schedules (typically 65–66 Gy in 30 fractions) are frequently used. Patients are managed as category 1 patients and RT should be completed on time.

Target volume definition is performed using a contrast-enhanced planning CT scan. Co-registration of the planning CT scan with the diagnostic MRI scan can aid target volume delineation. An anatomical (inclusion of the whole oropharynx) or geometric (inclusion of gross tumour volume with a defined margin) approach may be used for primary target volume delineation. Prophylactic RT should be given to the ipsilateral cervical lymph nodes for lateralised (e.g. tonsillar) tumours and to both sides of the neck for non-lateralised tumours (defined as tumours which involve greater than 1 cm of a midline structure e.g. soft palate and/or tongue base). Radiotherapy to levels II, III and IVa is recommended; level Ib may also be included in cases with anterior extension of tumour and/or involvement of the anterior tonsillar pillar. Planning can be carried out using three-dimensional conformal planning (typically using a ‘wedged pair’ of RT fields) or intensity modulated radiotherapy (IMRT) and/or Arc therapy.

**Recommendations**

• Treatment options for T1–T2 N0 oropharyngeal squamous cell cancer include: radical radiotherapy or transoral surgery and neck dissection (with post-operative (chemo)radiotherapy if there are adverse pathological features on histological examination) (R)
• Transoral surgery is preferable to open techniques and is associated with good functional outcomes in retrospective series (R)
• If treated surgically, neck dissection should include levels II–IV and possibly level I. Level IIb can be omitted if there is no disease in level IIa (R)
• If treated with RT, levels II–IV should be included, and possibly level Ib in selected cases (R)

## Advanced (T3–T4 N0 and T1–T4 N1–N3) oropharyngeal cancer

### 

#### General principles of management

A thorough review of the literature relating to the management of oropharyngeal cancer was published as a Cochrane report in 2009. The only evidence of statistically significant benefit was for the addition of concomitant chemotherapy to post-operative RT.^19^ All other treatment comparisons did not show any statistical differences.

In recent years, there has been a tendency to offer primary RT and/or chemoradiotherapy for oropharyngeal carcinoma, as part of an ‘organ preservation’ strategy. Although there are no good head-to-head comparisons of primary surgery and chemoradiotherapy for stage III/IV OPSCC, outcomes from randomised trials of chemoradiotherapy (e.g. RTOG 0129) are at least comparable to the results of surgical series. One potential concern with an organ preservation approach is that although salvage surgery has been shown to have a high success rate for laryngeal cancer, the success rate of salvage surgery is not the same in other head and neck sites, such as the oropharynx.

The 2013 National Head and Neck Cancer Audit (9th DAHNO Report) concluded that variation in treatment strategies for OPSCC is evident across cancer networks in England and Wales. This is not surprising in view of the fact that current published evidence does not provide a consensus view to define the most appropriate treatment strategy. Treatment decisions for individual patients will depend on the size, position and overall functional deficit, as well as on patient preference and local expertise. Human papilloma virus status has a profound influence on prognosis, and in future, could potentially affect selection of treatment modality. Recruitment into randomised controlled clinical trials addressing these issues is highly recommended.

#### Surgical management of advanced oropharyngeal carcinoma

Where facilities and expertise exist, transoral resection (by TLM or TORS) of base of tongue, tonsil and pharyngeal wall primary tumours (usually with post-operative (chemo)radiotherapy) has been shown to offer rates of cure which appear to be as good as primary chemoradiotherapy in non-randomised comparisons, with promising functional results. Transoral resection is generally restricted to T1–T2 tumours, although resection of some T3 tumours may be considered if it is anticipated that negative margins can be achieved via a transoral approach. Transoral resection is rarely appropriate for T4 primary tumours. Also, where a larger resection of the soft palate is required, the general consensus is that surgery gives a poor functional outcome. It should be noted that approximately 80 per cent of patients who undergo primary surgery will also receive post-operative RT or chemoradiotherapy.

If transoral resection is not appropriate, e.g. for large primary tumours, then chemoradiotherapy should be considered. Alternatively, open surgical procedures may be considered, which usually require paramedian mandibulotomy for access and reconstruction with a flap. Trans-cervical pharyngotomy alone can be used for tongue base resections. Other approaches, such as glossotomy and lingual release can be used but are not often employed. Reconstruction is generally performed using radial artery free flaps or anterolateral thigh free flaps. Reconstruction using pedicled flaps, such as pectoralis major should be considered sub-optimal. Functional results following open surgery can be poor, particularly when followed by adjuvant therapy.

There are several published case series that report the likelihood of nodal metastasis for advanced oropharyngeal carcinoma to be over 50 per cent. When managing T3 and T4 oropharyngeal cancers, the N0 neck should be treated electively. When managing the N0 neck surgically, a selective level II, III and IV neck dissection is generally recommended, and in some cases level I may be included. All patients with node positive disease should have a modified neck dissection or at least level I–IV selective neck dissection.

#### Primary chemoradiotherapy for loco-regionally advanced (stage III–IVb) oropharyngeal carcinoma

Chemoradiotherapy (organ preservation) is an effective treatment choice for advanced head and neck tumours. A RT dose equivalent of 70 Gy in 2 Gy fractions with concurrent cisplatin chemotherapy is considered standard for stage III and/or IV OPSCC. Concurrent weekly cetuximab (a monoclocal antibody targeting the epidermal growth factor receptor) may be given with RT if there is a contraindication to platinum chemotherapy (e.g. renal dysfunction or hearing impairment). Alternatively, radical RT alone can be given for patients with advanced disease who are not fit for concurrent treatment, particularly if they are over 70 years of age when the benefits of concurrent chemotherapy are reduced. Induction chemotherapy may be considered for patients with advanced (T4, N3, N2c) disease to reduce the risk of distant metastases^20^ and for selected other patients with bulky primary (T4) and/or nodal disease (N3), but there is currently no high-quality evidence of its efficacy in these indications.

The principles of RT outlining and planning are as described for earlier stage disease. Neck nodes should be included in the treatment fields depending on their probability of involvement and according to the DAHANCA, EORTC, HKNPCSG, NCIC CTG, NCRI, RTOG, TROG consensus guidelines and atlas which were updated in 2013.^21^ Radiotherapy to levels Ib–IVa, V(a,b) and the retropharyngeal nodes (level VIIa) at the level of the oropharynx is generally recommended in a node positive neck. The retrostyloid space (level VIIb) is included when level II is involved and the supraclavicular fossa (levels IVb and  Vc) is included when level IVa or V is involved. Radiotherapy should be given to at least the ipsilateral cervical lymph nodes for lateralised tumours and to both sides of the neck for non-lateralised tumours. The issue of whether the contralateral neck should be treated in patients with lateralised oropharyngeal tumours and advanced (N2+) nodal disease remains controversial and will depend on local practice.

Chemoradiotherapy is associated with greater toxicity than RT alone and late toxicity, particularly swallowing dysfunction, can have a significant impact on quality of life.^22^ Gastrostomy tube dependence rates of up to 24 per cent at 1 year and 14 per cent at 2 years post-chemoradiotherapy have been reported, although others have reported much lower rates. Improvements in RT techniques (including IMRT) have been shown to reduce late complications following RT. The UK PARSPORT randomised study showed a significant reduction in xerostomia rates with parotid sparing IMRT compared with conventional RT (using parallel opposed fields) in patients with advanced OPSCC.^23^ Ongoing studies are exploring the role of IMRT in improving swallowing function following RT, by reducing radiation dose delivery to the pharyngeal constrictor muscles and other swallowing structures.

Traditionally, patients with advanced nodal disease (N2 or N3) being treated by chemoradiotherapy required a planned neck dissection, with little evidence to support whether neck dissection before or after chemoradiotherapy is more effective. There is now level I evidence from the PET-Neck trial that a PET–CT guided active surveillance policy, with neck dissection only being carried out if residual abnormal or equivocal nodes are present on imaging 10–12 weeks after the end of chemoradiotherapy, results in similar survival rates to a planned neck dissection, with less morbidity, and with higher cost effectiveness.^5^

#### Post-operative radiotherapy and chemoradiotherapy for advanced oropharyngeal carcinoma

The indications for post-operative RT and chemoradiotherapy for OPSCC depend on pathological risk factors for recurrence common to most head and neck squamous carcinomas. Randomised controlled trials and a meta-analysis of results confirm that patients with extra-capsular invasion and/or microscopically involved (<1 mm) surgical resection margins around the primary tumour experience significant benefit in terms of overall and disease free survival from post-operative chemoradiotherapy compared with RT alone.^24^ Post-operative chemoradiotherapy is associated with significant acute and late toxicity and is not generally recommended in patients over 70 years of age and/or patients with poor performance status. Indications for post-operative RT alone include multiple nodal metastasis, T3 or T4 tumours, and tumours with other adverse features, including perineural or lymphovascular invasion. Patients with close (1–5 mm) surgical margins around the primary tumour may be treated with post-operative chemoradiotherapy or RT alone according to the presence or absence of other risk factors for recurrence. Patients should start their adjuvant RT as soon as possible after surgery (ideally within five weeks (35 days) and no later than six weeks (42 days)) to avoid reduced local control and survival due to protracted treatment.

The relevance of traditional risk factors for recurrence (including extra-capsular spread) and the benefit of adjuvant chemotherapy with RT in the context of HPV-positive OPSCC has been questioned by some studies. However, no change in management of patients should occur outside clinical trials. Clinical trials which aim to modify adjuvant treatment based on HPV status are currently ongoing in the UK and USA.

## Ongoing Research

Human papilloma virus status appears to have profound influence on prognosis and, in the future, potentially on selection of treatment modality. There are several ongoing or planned clinical trials for HPV-positive and HPV-negative OPSCC and recruitment into clinical trials addressing these issues is highly recommended. Development of biomarker classifiers for treatment selection is also high recommended.

**Recommendations**

• Advanced oropharyngeal carcinoma can be treated with primary chemoradiotherapy or transoral surgery and adjuvant (chemo)radiotherapy (R)
• The N0 neck should be treated electively – either by radiotherapy or selective neck dissection (R)
• Patients with advanced nodal (N2 or N3) disease receiving radical chemoradiotherapy should have a PET-CT scan 10–12 weeks after treatment, with a subsequent neck dissection within 4 weeks if residual abnormal or equivocal nodal disease is detected (R)
• Intensity modulated radiotherapy reduces toxicity in patients treated with radical radiotherapy, compared with conventional radiotherapy (R)
• Post-operative chemoradiotherapy is currently recommended in patients treated with surgery who have involved primary tumour resection margins and/or extracapsular spread of nodal disease. Otherwise, post-operative radiotherapy alone may be indicated (R)

### Key points

• Oropharyngeal cancer incidence is increasing rapidly in the UK due to the Human papillomavirus (HPV).
• HPV association confers better outcomes regardless of treatment modality
• Early stage disease should be receive single modality treatment
• Advanced disease should receive combined modality treatment
• PETCT scanning undertaken at 10-12 weeks post chemo-radiation results in similar survival to planned neck dissection, but with considerably fewer patients requiring neck dissection, less morbidity and is cost-effective
• There is insufficient evidence to alter treatment on the basis of HPV status
• Patients should be offered the opportunity to participate in the ongoing clinical trials.
